# Impact of sexual intercourse on frozen-thawed embryo transfer outcomes: a randomized controlled trial

**DOI:** 10.1186/s40834-023-00218-y

**Published:** 2023-03-02

**Authors:** Jin-Wei Hou, Li-Hua Yuan, Xian-Ling Cao, Jing-Yan Song, Zhen-Gao Sun

**Affiliations:** 1 The First Clinical College, Shandong University of Traditional Chinese Medicine, Jinan, China; 2 Reproductive and Genetic Center of Integrated Traditional and Western Medicine, The Affiliated Hospital of Shandong University of Traditional Chinese Medicine, Jinan, China

**Keywords:** Sexual intercourse, Barrier contraception, In vitro fertilization, Frozen thawed embryo transfer, Pregnancy outcomes

## Abstract

**Background:**

Exposure of the female reproductive tract to either seminal plasma or fluid component of the ejaculate is beneficial to achieving successful embryo implantation and normal embryo development. But whether the “physical” component of sexual intercourse during the peri-transfer period have any influence on frozen-thawed embryo transfer (FET) pregnancy outcomes is not clear.

**Methods:**

We conducted a randomized trial that included 223 patients undergoing in vitro fertilization (IVF) treatment at a university-affiliated reproductive center from 19 July 2018 to 24 February 2019. Enrolled patients undergoing IVF treatment were randomized either to engage sexual intercourse using the barrier contraception (Group A, *n* = 116) or to abstain (Group B, *n* = 107) one night before FET. The primary outcome was clinical pregnancy rate.

**Results:**

Patients having intercourse had higher clinical pregnancy rate (51.72% vs. 37.07%, *P* = 0.045) and implantation rate (38.31% vs. 24.77%, *P* = 0.005) compared to those did not engage intercourse. However, there was no significant difference of the spontaneous abortion rate between two groups (11.67% 33 vs. 14.63%, *P* = 0.662).

**Conclusions:**

Sexual intercourse before embryo transfer may improve the clinical pregnancy and implantation rates during FET cycles. However, it should be noted that patients choose only one time for sexual intercourse, that is, the night before embryo transfer.

**Trial registration:**

The present study was registered at the Chinese Clinical Trial Registry (http://www.chictr.org.cn/, ChiCTR1800017209).

## Background

In the process of natural sexual reproduction, the act of intercourse and presence of seminal fluid are essential factors for pregnancy. With respect to the latter, studies have shown that the soluble and exosome-born signaling agents present in the semen interact with the female reproductive tract and induce an immune response that may benefit the pregnancy outcome [1]. However, whether physical act of sexual intercourse has any beneficial effect on the chance of pregnancy during the cycle of assisted reproductive technology (ART) is still controversial. Studies have shown that sexual intercourse may cause subclinical upper reproductive tract infection that is associated with decreased implantation rates [2] as well as ascending uterine infection during third-trimester gestation [3]. Indeed, the uterus is very vulnerable to pathogens introduced by sexual intercourse because the cervical mucus barrier to ascending infection is disrupted by mechanical passage of the catheter during the embryo transfer procedure of IVF treatment. Moreover, the act of intercourse may induce the event of orgasm that increases the activity of uterine myometrium [4]. Therefore, the intercourse-induced increases in levels of uterine activity are highly correlated with uterine contractions that may be beneficial for embryo implantation and affect IVF outcome [5].

On the other hand, sexual intercourse has beneficial effects on assisted implantation. Studies performed using animal models revealed that exposure of the female reproductive tract to either seminal plasma or fluid component of the ejaculate is beneficial to achieving successful embryo implantation and normal embryo development [6–8]. For instance, rodent studies performed using artificial insemination or embryo transfer with being exposed to seminal plasma have substantially higher pregnancy rates than those without being exposed [6, 7]. Similarly, rat embryos inseminated with spermatozoa before blastocyst transfer have a higher implantation rate than those without spermatozoa exposure [9]. Additionally, increasing evidence suggests that women who have tissue contact with husband’s semen during ART treatment improve their pregnancy outcomes [10–12]. A meta-analysis study performed using seven randomized controlled trials that involved 2,204 patients showed that women with either sexual intercourse or passive exposure to semen around the time of IVF treatment had an increase in clinical pregnancy rate (approximately 23%) [11]. Collectively, these findings indicate that components in the semen may induce an immune response that may positively affect the endometrial receptivity and pregnancy outcomes.

Interestingly, sexual arousal has been shown to improve the motility of spermatozoa, although the detailed molecular mechanism is not clear [13]. Furthermore, studies revealed that patients with sexual intercourse without contraception had a higher incidence of the risk of life-threatening complication as a heterotopic pregnancy as a consequence of spontaneous pregnancy after sexual intercourse during their FET cycles [14]. At present, it remains unclear whether the beneficial effects of sexual intercourse on the endometrial receptivity and embryo implantation are due to the transportation of semen into the vagina or the sexual behavior itself. Therefore, we aimed to investigate whether the act of sexual intercourse itself (using condoms as contraception) has any influence on clinical pregnancy rates in patients with IVF treatment.

## Methods

### Study design

This is a unblinded randomized study conducted at the Center for Reproduction and Genetics of the Affiliated Hospital of Shandong University of Traditional Chinese Medicine (TCM). This study was approved by the University Research Ethics Committee and registered at the China Clinical Trial Registration Center (ChiCTR1800017209). All participants signed an informed consent form prior to the study enrollment.

### Study participants

A total of 300 infertile patients undergoing FET were recruited from the Center for Reproduction and Genetics of the Affiliated Hospital of Shandong University of TCM, from 19 July 2018 to 24 February 2019, during this period, patients undergoing embryo transfer will be included after signing informed consent.

Inclusion criteria were as follows: 1) at least one cycle of IVF/intracytoplasmic sperm injection (ICSI) performed in our reproductive center; 2) female age ≤ 35 years; 3) a body mass index (BMI) between 18.5 kg/m^2^ and 25 kg/m^2^; 4) regular menstrual cyclicity (24–35 days); 5) the attending physician for embryo transfer being the same person for all patients; 6) the tubal factor is the cause of female infertility( bilateral tubal obstruction, or hydrosalpinx treated using laparoscopic surgery); 7) Four or more embryos available for transfer with at least one viable day 3 embryo with a score of 6 or above according to the Istanbul Consensus [15] and without receiving preimplantation genetic screening (PGS) or preimplantation genetic diagnosis (PGD).

Exclusion criteria were as follows:1) patients with known congenital uterine malformation, uterine fibroids, or endometriosis; 2) chromosomal abnormalities in any of infertile couples; 3) female with immune diseases.

### Randomization

Patients are randomly grouped according to the random list generated by the computer. The exact time of randomization was on the day of endometrial transformation. Randomization via website (www.randomization.com). The online randomization program was operated by a special data expert, who did not participate in patient recruitment and clinical management. The person then prepares the randomly grouped cards and puts them in an opaque envelope. On the day of randomization, the subjects were randomly divided into two groups according to the opaque envelope. Group A was subjected to have intercourse using a condom at the night before embryo transfer, while group B was subjected to abstain for the entire IVF cycle. Signed written informed consents were obtained from all participants before the study. Information about whether patients engage sexual intercourse using the barrier contraception or to abstain one night before FET is sealed in an opaque envelope with only one number on it. In order to prevent patients from not following the doctor’s orders, we will contact the patients and their spouses again according to the results after unblinding to confirm that the content provided is true.

### Interventions

In the present study, artificial endometrium preparation by the exogenous administration of estrogen and progesterone was used for the FET cycle. To prepare the endometrium, all patients self-administered oral estradiol valerate (France; DELPHARM Lille S.A.S.) 8 mg per day started at the third to fourth day of the menstrual period for 5 days, and then 12 mg per day for the remaining days of the cycle, which was adjusted according to the clinical situation during this period. Estrogen administration was continued until the endometrium reaches a thickness of 8 mm (determined by an ultrasonographic examination). And then progesterone was combined to initiate the secretory phase in preparation for FET. Two vitrified-warming embryos were selected for transfer on the fourth day of progesterone administration based on morphological grading according to the Gardner and Schoolcraft scale. Luteal support was routinely provided after FET for 14 days irrespective of pregnancy. For patients confirmed with a clinical pregnancy, estradiol valerate and progesterone were continued until 10 weeks of gestation, which was gradually reduced until detecting the fetal heart beats.

### Participant follow-up and data collection

The primary outcome was clinical pregnancy rate, which was defined as the detection of fetal heart beats using ultrasonographic examinations 35 days after FET. Serum beta subunit of human chorionic gonadotropin (β-hCG) levels were examined using electrochemiluminescence analysis (Cobas e411 System Product, Germany) on day 14 following embryo transfer in all patients. Implantation was defined as a serum quantitative β-hCG ≥ 10 U/L. All pregnancies were followed until 7 weeks after FET. A biochemical loss is defined as a transient but significant increase in serum β-hCG ≥ 10 U/L between days 12 and 20 after embryo transfer without detecting a gestational sac by an ultrasonography. An early abortion is defined as the detection of an empty gestational sac using ultrasonographic examinations before 12 weeks of gestation. Implantation rate is defined as the ration of the number of embryos implanted and the number of transferred embryos.

### Sample size calculation

The sample size calculation was done based on previous studies, in which the efficacy of sexual intercourse during the peri-transfer period was 24% [10], α = 0.05 and β = 0.2. According to the ratio of 1:1, 10% of the lost visit rate, there were expected to be 104 patients in the barrier contraception group and 104 patients in the abstain group.

### Statistical analysis

The statistical software package, IBM SPSS Statistics for Windows, version 22.0 (IBM Corporation, Armonk, NY, USA), was used for all data analyses. The data are expressed as mean ± standard deviation (x ± s). Among those were normally distributed and homogeneity of variance (homoscedasticity) was satisfied, a *t* test was performed; otherwise, Mann–Whitney U test was performed. The treatment outcomes for each group were counted and expressed as a frequency. When the total sample size was greater than 40 and the minimal theoretical frequency was greater than 5, we applied the Chi-square test. When the total sample size was less than 40 or the minimal theoretical frequency was less than 5, we applied the Fisher exact-probability method. *P* < 0.05 was considered statistically significant. The intention to treat analysis will not be performed because we had only one intervention after randomization and did not use any experimental drugs.

## Results

**Fig. 1:**
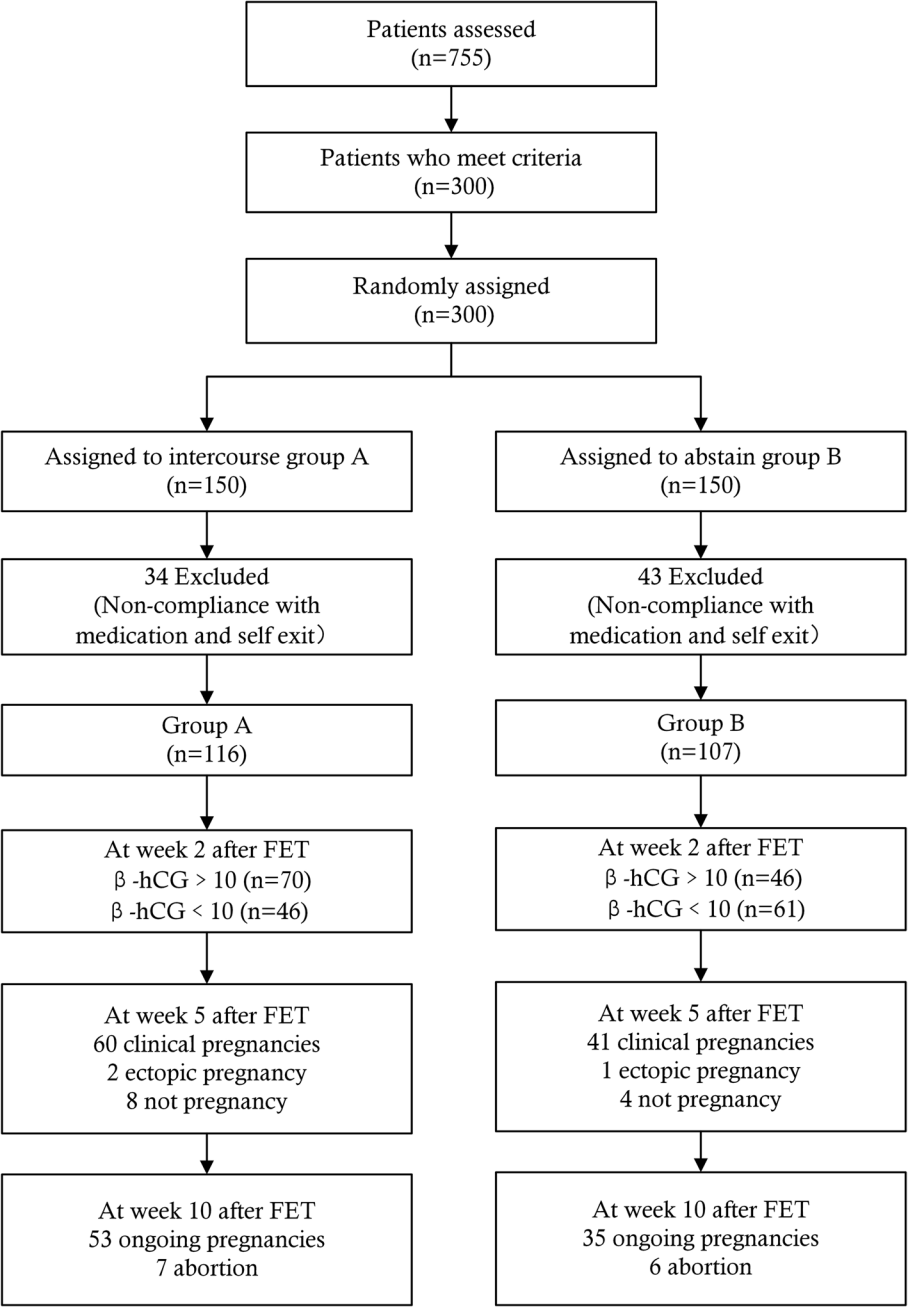
Flow chart of individuals selection

Three hundred patients requiring IVF treatment were randomly divided into two groups, with 150 patients in each group. Group A (*n* = 150) was subjected to have intercourse using a condom at the night before embryo transfer, while group B (*n* = 150) was subjected to abstain for the entire IVF cycle. Patients engaged in intercourse one night before embryo transfer using a barrier contraception (condoms). All patients participating in the study underwent vaginal scrubbing prior to embryo transfer. Owing to non-compliance with medication and personal concerns resulting in cycle cancelation. Ultimately, 223 patients were included in the analysis, with 116 cases in group A and 107 cases in group B (Fig. 1). The characteristics of trial participants were similar, confirming the adequacy of the randomization process. No significant difference in major parameters between the two groups, including maternal age (30.27 ± 3.58 vs. 29.83 ± 3.05), BMI (22.04 ± 2.32 vs. 22.48 ± 1.81), average FSH concentrations (6.54 ± 1.79 vs. 6.94 ± 1.77), duration of infertility (2.33 ± 1.40 vs.2.64 ± 1.33), number of good-quality embryos (3.10 ± 1.35 vs. 2.79 ± 1.19), gravidity (1.19 ± 1.26 vs. 1.27 ± 1.36), parity (0.41 ± 0.53 vs. 0.37 ± 0.52), nulliparous (42.24% [49/116]) vs. 42.99% [46/107]), history of spontaneous abortion (20.69% [24/116) vs. 17.76% [19/107]), and endometrial thickness on the day of FET (mm) (10.50 ± 1.55 vs. 10.53 ± 1.45), (Table 1).

**Table 1:** Clinical characteristics for enrolled patients undergoing frozen-thawed embryo transfer

	Group A	Group B	P
No. of cycles (n)	116	107	
Age (years)	30.27 ± 3.58	29.83 ± 3.05	0.331
BMI (kg/m^2^)	22.04 ± 2.32	22.48 ± 1.81	0.116
bFSH (IU/L)	6.54 ± 1.79	6.94 ± 1.77	0.103
Duration of infertility (years)	2.33 ± 1.40	2.64 ± 1.33	0.103
Gravidity	1.19 ± 1.26	1.27 ± 1.36	0.643
Parity	0.41 ± 0.53	0.37 ± 0.52	0.572
Nulliparous	42.24% (49/116)	42.99% (46/107)	0.910
History of spontaneous abortion	20.69% (24/116)	17.76% (19/107)	0.579
No. of high-quality embryos (n)	3.10 ± 1.35	2.79 ± 1.19	0.072
Endometrial thickness on FET day (mm)	10.50 ± 1.55	10.53 ± 1.45	0.883

Group A: patients were engaged to have sexual intercourse; Group B: patients were engaged to abstain

Data are expressed as means ± variance (s)

*BMI* Body mass index, *bFSH* basal follicle-stimulating hormone, *SET* Single embryo transfer, *DET* Double embryo transfer

Notably, a significant difference in the clinical pregnancy rate (51.72% [60/116] vs. 38.31% [41/107], *P* = 0.045) was present between the two groups (Table 2). Similarly, the implantation rate was significantly higher in group A than in group B (37.07% [86/232], vs. 24.77% [53/214], *P* = 0.005) (Table 2). However, no significant difference in the biochemical losses rate was present between two groups (11.43% [8/70] vs. 8.7% [4/46], *P* = 0.761) (Table 2). Similarly, although few case numbers, there was no significant difference of the spontaneous abortion rate between two groups (11.67% [7/60] vs. 14.63% [6/41]), *P* = 0.662) (Table 2).

**Table 2:** Comparison of pregnancy outcomes between the two study groups

	Group A	Group B	P
Clinical pregnancy rate	51.72% (60/116)	38.31% (41/107)	0.045
Implantation rate	37.07% (86/232)	24.77% (53/214)	0.005
Biochemical losses rate	11.43% (8/70)	8.70% (4/46)	0.761
Spontaneous abortion rate	11.67% (7/60)	14.63% (6/41)	0.662

## Discussion

Assisted reproductive technology has evolved rapidly over the past 30 years, which is as frequent for conceiving a child as any other minor medical procedure. Despite the recent advances in IVF, the successful rates (clinical pregnancy and subsequent live birth rates) remain disappointingly low because of the multiple causes of infertility, individual patient differences, and intolerance to embryo transfer. Among these multiple factors, the implantation rate is limited despite the transfer of excellent-quality embryos. Indeed, repeated failures of implantation due to inadequate uterine receptivity has become a compelling challenge in reproductive medicine. A meta-analysis revealed a statistically significant improvement in clinical pregnancy rate when women are exposed to seminal plasma (unprotected sexual intercourse, intra-vaginal, intracervical, or intrauterine application) around the time of oocyte pick-up or embryo transfer [11]. Studies performed using animal models and human samples suggest an immune-regulatory role for seminal plasma in embryo implantation [11]. But at present, no trial study has investigated whether the “mechanical” act of sexual intercourse before the time of embryo transfer has any influence on implantation and clinical pregnancy.

The results obtained from this randomized study are the first to provide evidence that the act of sexual intercourse (excluding the effect of semen plasma) during the time of embryo transfer is not detrimental to implantation or pregnancy outcome. Furthermore, female patients having sexual intercourse exhibited substantial improvement in both implantation and pregnancy rates. All patients underwent artificial cycle FET. Therefore, natural conception of the enrolled patients in the sexual intercourse group was unlikely to occur.

The observed findings that sexual intercourse had a beneficial effect on pregnancy outcome may be explained by several factors. The physical act of sexual intercourse arousal has been shown to increase the blood flow of female genital tract [16], which could enhance the blood supply of the endometrium, resulting in improvement of endometrial receptivity. Indeed, increasing evidence suggests that adequate uterine perfusion contributes to the uterine receptivity, and an increased impedance in uterine flow is associated with infertility [17, 18]. Future studies are required to investigate the distribution of endometrial blood flow and the impedance changes in uterine artery blood flow before and after sexual intercourse. Previous studies also showed that sexual intercourse can activate various female genital tract reflexes, which may further induce several beneficial effects, such as increased production of sex hormones and maintenance of general tract functionality [13]. The other potential beneficial impact of sexual intercourse on the pregnancy outcome is that coitus is considered as a type of behavioral treatment approach that might be efficacious for emotional aspects of infertility. A study performed including 54 infertile patients showed that behavior treatment significantly decreased degree of anxiety, depression, and fatigue, but increased vigor, of which 34% of these women became pregnant later on [19]. Therefore, if the act of sexual intercourse can make women less stressful, it may, to some extent, reduce the anxiety of patients perceived prior to embryo transfer. Moreover, encouraging couples to engage in sexual intercourse during their IVF treatment gains an additional psychological advantage, as they might feel more natural and comfortable during the process of conception. Certainly, all these proposed mechanisms are issues worth exploring further.

Of course, there are some limitations to this study. For example, it is important to note that our selected time of coitus for patients was only once, the night before the embryo transfer. Our interpretation of the results obtained in the present study has been to brief an interval to show beneficial effects. It is difficult to determine which day is the best time point for sexual intercourse before embryo transfer. However, a study performed using animal models showed that the pregnancy rates after artificial insemination in ewes were much lower than those after natural mating, although the mechanism remains unclear [20]. Therefore, the results obtained from animal studies suggest that the act of natural mating does not appear to increase uterine activity and subsequent pregnancy rate. Future studies aimed at addressing these issues by conducting a study design with various time points of sexual intercourse to investigate the effects on subsequent pregnancy outcomes. Besides, limitations of our study also include the non-blindness and small sample size which will reduce the persuasiveness of our study. Additionally, it is important to note that we have failed to investigate the potential effects of various sexual intercourse behaviors, including penis, fingers, and sex toys, as well as the duration of sexual intercourse, whether orgasm occurs, or subjective sexual satisfaction on the outcome. Moreover, only heterosexual patients were included in this study, which was not considered for lesbian partners. Therefore, the beneficial population for the results of this study still needs to be interpreted with caution.

## Conclusions

In summary, clinical data obtained from this study demonstrate that the “physical” component of sexual intercourse before the day of embryo transfer may improve the clinical pregnancy and implantation rates during FET cycles. However, there is much work to be done in investigating the functional relationships and the underlying mechanisms to support the theoretical basis. A large-sample, multicenter, randomized controlled trial study is therefore needed to confirm the effects of sexual intercourse on IVF outcomes.

## Data Availability

Data and material are available with the corresponding author.
